# 

**Published:** 1805-09-01

**Authors:** 


INDEX TO THE FIRST SERIES OF THE MEDICAL AND PHYSICAL JOURNAL.

*The numerous friends and purchasers of the Medical and Physical Journal, are respectfully informed, that it is intended to prepare and publish, after the completion of the* TWENTIETH VOLUME, *and of every future twentieth volume, a very full and complete Index to the preceding series; such Index to include separate references to the Diseases, Remedies, and Writers.*

*Prefixed will be given a general History of Medicine, Surgery, and the Physical Sciences, during the period of the publication of the twenty volumes, with particular reference to the valuable communications of the Correspondents of the Medical and Physical Journal.*

*It is supposed that the Historical Introduction and the Index will, together, form a volume nearly equal in size to an ordinary volume of the Journal, and perhaps not inferior in value, as a body of useful reference, to any medical work in the English language.*

---

### To CORRESPONDENTS.

Communications are received from Mr. Dunning, Mr. Wadley, Mr. Weston, Cantab, and S. M.

